# Mechanism underlying the carotenoid accumulation in shaded tea leaves

**DOI:** 10.1016/j.fochx.2022.100323

**Published:** 2022-05-04

**Authors:** Xiumin Fu, Jiaming Chen, Jianlong Li, Guangyi Dai, Jinchi Tang, Ziyin Yang

**Affiliations:** a Guangdong Provincial Key Laboratory of Applied Botany & Key Laboratory of South China Agricultural Plant Molecular Analysis and Genetic Improvement, South China Botanical Garden, Chinese Academy of Sciences, No. 723 Xingke Road, Tianhe District, Guangzhou 510650, China; b University of Chinese Academy of Sciences, No. 19A Yuquan Road, Beijing 100049, China; c Tea Research Institute, Guangdong Academy of Agricultural Sciences & Guangdong Provincial Key Laboratory of Tea Plant Resources Innovation and Utilization, No. 6 Dafeng Road, Tianhe District, Guangzhou 510640, China; d Center of Economic Botany, Core Botanical Gardens, Chinese Academy of Sciences, No. 723 Xingke Road, Tianhe District, Guangzhou 510650, China

**Keywords:** α-Carotene (PubChem CID: 4369188), β-Carotene (PubChem CID: 54603957), *δ*-Carotene (PubChem CID: 5281230), Biosynthesis, *Camellia sinensis*, Carotenoids, Metabolic regulation, Tea, Shade

## Abstract

Carotenoids contribute to tea leaf coloration and are the precursors of important aromatic compounds. Shading can promote the accumulation of carotenoids in tea leaves, but the underlying mechanism remains unknown. In the study, we analyzed the content and composition of carotenoids, and transcript levels and functions of related genes in carotenoid biosynthesis using HPLC, qRT-PCR, and heterologous expression system. It was found that long-term shading (14 days, 90% shading) significantly increased the total carotenoid content in tea leaves, and increased the expression of non-mevalonate pathway (MEP) genes (*CsDXS1* and *CsDXS3*) and key genes in carotenoid synthesis pathway (*CsPSY*, *CsLCYB*, and *CsLCYE*). Long-term exposure to darkness (14 days, 0 lx) decreased the transcription of most carotenoid biosynthetic genes and adversely affected carotenoid accumulation. Furthermore, CsDXS1, CsDXS3, CsPSY, CsLCYB, and CsLCYE were functionally identified and contributed to the enhanced accumulation of carotenoids in tea leaves in response to long-term shading.

## Highlights

• Long-term shading treatment (14 days) increased carotenoid content in tea leaves.
• Long-term darkness (14 days) decreased carotenoid content in tea leaves.
• Long-term shading treatment increased carotenoid biosynthetic gene expression levels.
• Long-term darkness decreased carotenoid biosynthetic gene expression levels.
• The functions of *CsDXS1*, *CsDXS3*, *CsPSY*, *CsLCYB* and *CsLCYE* genes have been verified.

## 1 Introduction

In China, tea plant (*Camellia sinensis*) is a crucial economic crop. The quality of tea leaves is the main factor influencing the economic value of tea products. Many characteristic metabolites, such as amino acids, polyphenols, aromatic compounds, and pigments, affect tea quality (Wan, 2015). More specifically, carotenoids are important for tea leaf coloration, while also serving as the precursors of crucial tea aromatic compounds (e.g., β-ionone and α-ionone). Carotenoids are degraded into floral aromatic compounds under enzymatic or non-enzymatic reactions, especially during tea manufacturing (Fu, Tang, & Yang, 2021). Previous studies revealed that the content and composition of carotenoids and their degradation products are determinants of tea quality (Fu et al., 2021, Ravichandran, 2002). Therefore, elucidation of tea carotenoid biosynthetic and regulatory mechanisms may provide researchers with important information to improve tea quality. Carotenoid biosynthesis and regulation have mainly been investigated in the flowers, fruits, and roots of various species during ripening or the post-harvest stage (Ngamwonglumlert, Devahastin, Chiewchan, & Raghavan, 2020). Carotenoid research involving green plants, such as *Arabidopsis thaliana*, has mainly paid attention to the roles of carotenoids on photosynthesis, growth, and hormone regulation (DellaPenna, 1999).

Earlier research revealed that different plants vary in terms of their strategies for adapting to shade stress. Specifically, plants may be divided into two categories, including shade-avoiding and shade-tolerant plants. When some shade-avoiding plants, such as *A. thaliana*, are exposed to light with a low red:far-red ratio, their shade-avoidance mechanisms may be triggered to promote stem elongation, decrease branching, enhance reproductive growth, and modulate photosynthetic metabolism (e.g., decrease chlorophyll and carotenoid contents) (Franklin, 2008). However, this shade-avoidance response does not occur in shade-tolerant plants (e.g., begonias growing under a tropical forest canopy, *Geranium robertianum*, *Picea abies*, and *Cardamine* species) under shade stress conditions (Gommers et al., 2017, Jacobs et al., 2016, Molina-Contreras et al., 2019, Ranade et al., 2019).

There have been relatively few studies on the regulation of carotenoids in tea leaves. Similar to the carotenoid regulation in other plants (Fu et al., 2019), it was demonstrated that light also influences the synthesis and accumulation of tea pigments (Yue, Wang, & Yang, 2021). The shading of commonly grown green tea varieties will lead to the increased intensity of green coloration on leaves (Chen et al., 2021). Sonobe, Miura, Sano, and Horie (2018) used different shading treatments (0%, 35%, 75%, and 90%) and observed that the carotenoid content increases as the degree of shading increases. Additionally, carotenoid accumulation can positively affect tea product flavors after processing (Sonobe et al., 2018). For example, Japanese Kabuse-cha tea has a characteristic tea fragrance (i.e., ‘ooika’). It has been revealed that ‘ooika’ may be the result of a carotenoid-derived aromatic compound (Kawakami, 1981). The researchers found that Kabuse-cha tea is produced from fresh tea leaves that have been shaded, which possibly resulted in an increase in the contents of carotenoids and the aromatic compounds derived from carotenoids (Kawakami, 1981).

Previous studies revealed that shading can promote the accumulation of carotenoids in tea leaves (Sonobe et al., 2018, Yue et al., 2021), but the underlying regulatory mechanism is still unclear. In this study, shading treatment in tea gardens and dark treatment indoors was applied. By analyzing carotenoid contents and expression levels of carotenoid biosynthetic genes, and conducting subcellular localization, prokaryotic expression-based functional verification, and tobacco transient overexpression experiments, we elucidated the molecular basis of carotenoid accumulation in shaded tea plants. The aim of this study was to elucidate the effects of light on carotenoid accumulation and underlying mechanism in tea leaves and to offer useful information to modify tea cultivation practices to enhance tea quality.

## 2 Materials and methods

### 2.1 Plant materials, treatments, and reagents

To investigate the effect of shading on carotenoids accumulation in tea plants, fifteen-year-old tea plants (*C. sinensis* cv. Jinxuan) (23^o^N, 113^o^E, Yingde town, Guangdong province, China) were used as the research material. Light treatments were shown in the following parameters: CK, without shading treatment (daily average light intensity: 80,400 lx), 90% shading treatment (T1, daily average light intensity: 6544 lx), 98% shading treatment (T2, daily average light intensity: 1379 lx), and 99.5% shading treatment (T3, daily average light intensity: 263 lx). Meantime, the temperature and humidity of these treatments were detected (Fig. S1). After treatment with 7 days and 14 days, tea shoots containing one bud and two leaves from these tea plants were sampled to analyze the carotenoid contents and expression levels of related genes. The experiments were carried out in August 2020. Five tea shoots were collected as one biological repeat and three biological repeats were prepared.

To explore the effect of dark on carotenoids accumulation in tea plants, two-year-old tea seedlings (*C. sinensis* cv. Jinxuan) were exposed to the following treatments for 14 days, including treatment in a continuous dark environment (0 lx, 75% humidity, 25 °C), and control group with light/dark environment (16 h/8h, light intensity: 4000 lx, 75% humidity, 25 °C). Five tea shoots (each shoot with one bud and three leaves) from these tea seedlings were collected as one biological repeat and three biological repeats were prepared.

All biochemical, standards, kits, and reagents were molecular biology or cell culture grade from Sigma-Aldrich Company (USA) and Merck (China) unless otherwise stated.

### 2.2 Analysis of carotenoid content in tea leaves

Tea leaf samples (0.05 g) were extracted by methanol/H_2_O/chloroform (1:1:2) solvent. After centrifugation (10,000 *g*, 4 °C) for 10 min, the chloroform phase was collected. The content and composition of carotenoid was measured by high performance liquid chromatography (HPLC). HPLC analysis was operated on Waters e2695 (Waters Corporation, Milford, MA, USA) consisting of a 2998 photodiode array detector. The carotenoids in the extract (10 μL) were separated on YMC reverse-phase C_30_ (5 µm, 4.6 × 250 mm) column. 100% Methanol, 80% methanol containing 0.2% ammonium acetate, and methy-*tert*-butyl ether were applied as mobile phases in a gradient mode (1 mL min^−1^) (Fu et al., 2013, Fu et al., 2019). Chromatography was carried out at 25 °C and compounds were detected at 450 nm and quantified according to their respective standard curves.

To determine the total carotenoid contents in tea leaves was using a previously reported method (Lichtenthaler & Wellburn, 1983). Briefly, 80% acetone as the solvent to extract the total carotenoids at 4 °C in the dark, and the absorbance of the acetone extracts was measured at 663, 646, and 470 nm using a UV–Vis spectrophotometer. Total carotenoid contents were calculated as the below equations:

Chlorophyll *a* = 12.21 × A_663_ − 2.81 × A_646_;

Chlorophyll *b* = 20.13 × A_646_ − 5.03 × A_663_;

Carotenoids = (1000 × A_470_ − 3.27 × Chlorophyll *a* − 104 × Chlorophyll *b*)/229.

### 2.3 Expression level analysis of genes related to carotenoid biosynthesis

Total RNA of tea leaf sample was extracted using a Quick RNA isolation Kit, and 1 μg of them was reversely transcribed into cDNA using PrimeScriptTM RT reagent Kit. The primers of genes used for quantitative real-time PCR (qRT-PCR) analyses were shown in Table S1. The qRT-PCR analyses were performed on a Roche LightCycler 480 (Roche Applied Science, Mannheim, Germany) with the reactions in a total of 20 μL (0.5 μL of each primer (10 μM), 2 μL of cDNA template, 10 μL of iTaq Universal SYBR Green Supermix, and 7 μL of ddH_2_O). The qPCR system uses a program initiated with a preliminary step of 30 s at 95 °C, followed by 40 cycles at 95 °C for 5 s and 60 °C for 1 min. Change in mRNA level of target genes for each treatment was normalized to that of *encoding elongation factor 1-alpha* (*CsEF-1α*) (Forward primers, GTGTGGAGAAGAAGGACCCA; Reverse primers, CGAGGCTAGTGAACAGCAAC).

### 2.4 Full-length sequence cloning

The full-length sequences of *CsDXS1*, *CsDXS3*, *CsPSY*, *CsLCYB* and *CsLCYE* were cloned by referring to the NCBI and Tea Plant Information Archive (TPIA) database (Wei et al., 2018). The primers (Table S2) were designed using cDNA as template. The PCR system was 20 μL. The PCR reaction was predenatured at 98 °C for 30 s, denatured at 98 °C for 10 s, annealed at 58 °C for 15 s, extended at 72 °C for 60 s and 38 cycles, and extended at 72 °C for 1 min. The PCR results were detected with 1% agarose gel, the target strip gel was recovered, and the recovered product was sent to the company (Sangon Biotech (Shanghai) Co., Ltd., China) for sequencing. After sequencing, the sequences of these genes were aligned to the sequence in the TPIA database to ensure sequence correctness.

### 2.5 Transient expression in *Arabidopsis* protoplasts

The subcellular localizations of CsDXS1/DXS3/LCYB/LCYE/PSY were predicted by TargetP. The open reading frame (ORF) of *CsDXS1/DXS3*/*LCYB*/*LCYE*/*PSY* genes were subcloned into the pSAT6-EYFP-N1 vector (Table S3) according to the published method (Fu et al., 2021). More detailed information on *Arabidopsis* protoplast isolation and plasmid transformation methods were according to a previous publication (Fu et al., 2021).

### 2.6 Functional identification using *Escherichia coli* expression system

The plasmid pAC-LYC was used to test the lycopene ε-/β-cyclase activity (Cunningham, Sun, Chamovitz, Hirschberg, & Gantt, 1994). A lycopene-accumulating strain was used for heterologous complementation to test the function of *CsLCYE* and *CsLCYB.* The plasmid pAC-85b was used for testing PSY activity (Cunningham & Gantt, 2007). *CsLCYE*, *CsLCYB*, and *CsPSY* were amplified using primers shown in Table S4. The obtained cDNAs were subcloned into y2 plasmid digested (deletion of *A. thaliana LCYE* gene), and renamed pCsLCYE, pCsLCYB, and pCsPSY, respectively.

Chemically competent pAC-LYC-containing *E. coli* DH5α cells were prepared and transformed with pCsLCYB and pCsLCYE. Chemically competent pAC-85b-containing *E. coli* DH5α cells were prepared and transformed with pCsPSY. A single colony was cultivated in Luria Broth culture at 37 °C and 180 cycles per min. One mL aliquot of an overnight culture was added into 100 mL of LB medium. The cultures were grown for a further 48 h with shaking at 200 cycles per min and 30 °C under darkness. The cells were harvested by centrifugation at 5,000 *g* for 10 min and subjected to the analysis of carotenoid content as shown in the method Section 2.2 (Fu et al., 2019).

### 2.7 Functional identification using transient overexpression in tobacco

The ORFs of *CsDXS1*, *CsDXS3*, *CsLCYB*, *CsLCYE*, and *CsPSY* were cloned from tea cDNAs (Table S5). The method of transient overexpression in tobacco was the same as the previous description (Fu et al., 2021). The confirmed ORFs were recombined into the pCAMBIA3300 vector. The obtained vectors were transformed into *Agrobacterium* GV3101, followed by overnight cultivation. The cultures were collected and resuspended in a solution (pH 5.6; 10 mM morpholineethanesulfonic acid and MgCl_2_, and 100 μM acetosyringone) to OD_600_ = 0.8. The solution was infiltrated into *Nicotiana benthamiana* leaves using a syringe without a needle. The leaves were harvested at around 10:00 am after 6 days and subjected to the analysis of carotenoid content as shown in the method Section 2.2 (Fu et al., 2019).

### 2.8 Statistical analysis

One-way analysis of variance was performed on SPSS Statistics Software to test whether any differences existed among the data for gene relative expression levels or carotenoid contents in different treatments, and a *P*-value less or equal than 0.05 was regarded as significant.

## 3 Results

### 3.1 Long-term shading treatment increased the carotenoid accumulation in tea leaves

**Fig. 1:**
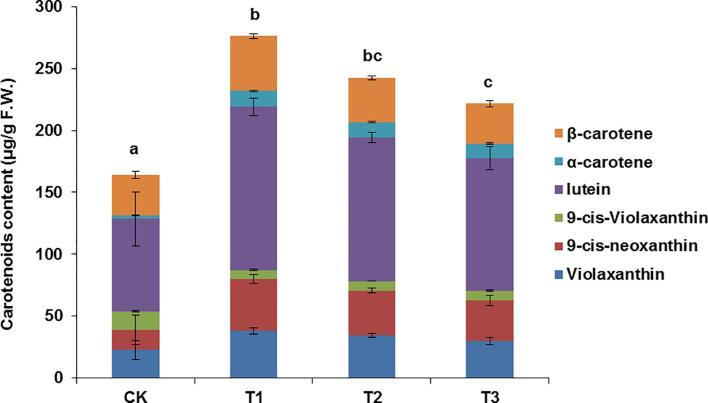
Carotenoid content and composition analysis of tea leaves under shading treatment for 14 days. CK, in nature light; T1, under 90% shading treatment; T2, under 98% shading treatment; T3, under 99.5% shading treatment. F.W., fresh weight. Data show the sum of individual carotenoid levels and are expressed as means ± S.D. (n = 3). Different letters above bars indicate significant differences using Duncan’s tests (*P* ≤ 0.05).

Lutein, β-carotene, 9-*cis*-neoxanthin, violaxanthin, and α-carotene were the main carotenoids detected in tea leaves. The carotenoid contents and compositions in tea leaves in response to different shading treatments are presented in Fig. 1. After 14 days of shading, the contents of most carotenoids increased significantly (Fig. 1). Compared with the control group (without shading treatment), the total carotenoid contents of tea plants that underwent the 90% and 98% shading treatments significantly increased by 1.69-times and 1.48-times, respectively (Fig. 1). Total carotenoid contents showed significantly higher in the tea leaves under 90% and 98% shading treatments than in the control group after 7 days of treatment, but the leaf carotenoid contents were only slightly higher in the shaded plants than in the control plants (about 1.15-times) (Fig. S2). Thus, the higher accumulation of carotenoids in shaded tea plants requires some time.

Carotenoid synthesis in plants is mainly mediated by the non-mevalonate (MEP) pathway, with DXS (1-deoxy-d-xylulose-5-phosphate synthase) responsible for the first and rate-controlling step of the pathway (Rohmer, Seemann, Horbach, Bringer-Meyer, & Sahm, 1996). A search of the Tea Genome Database (TPIA) detected four *DXS* homologs in tea, which were named *CsDXS1* (TEA008876), *CsDXS2* (TEA026768), *CsDXS3* (TEA012941), and *CsDXS4* (TEA012756). Phytoene synthase (PSY) is responsible for the first and rate-limiting reaction of the carotenoid synthesis pathway (Fray and Grierson, 1993, Fu et al., 2013). Two PSY-encoding genes (TEA022104 and TEA029725) were detected in TPIA. The tissue transcriptional data in the database indicated TEA029725 is mainly expressed in the roots, although it can also be expressed in other tissues. In contrast, TEA022104 is expressed in all tissues, but especially in flowers. Therefore, TEA022104 (designated as *CsPSY*) was selected as a candidate gene responsible for carotenoid synthesis in tea leaves. Lycopene ε-cyclase (LCYE) cyclizes lycopene to produce δ-carotene. Only one LCYE-encoding gene (TEA013706; *CsLCYE*) was identified in TPIA. Lycopene β-cyclase (LCYB) cyclizes lycopene to produce β-carotene, while also cyclizing δ-carotene to produce α-carotene. Only one LCYB-encoding gene (TEA012946; *CsLCYB*) is included in TPIA. Zeaxanthin is produced from β-carotene in a reaction catalyzed by β-carotene hydroxylase (BCH). The screening of TPIA detected only one BCH-encoding gene (TEA031348; *CsBCH*). Zeaxanthin is converted to violaxanthin by zeaxanthin cyclooxygenase (ZEP). Five ZEP-encoding genes were identified in TPIA, but the tissue-specific gene expression data indicated that only TEA013909 is highly expressed in tea leaves. Accordingly, this gene (*CsZEP*) was selected for further analysis. Lutein is generated from reactions catalyzed by BCH and ε-carotene hydroxylase (LUT). The one LUT-encoding gene (TEA014070; *CsLUT*) in TPIA was analyzed in this study.

**Fig. 2:**
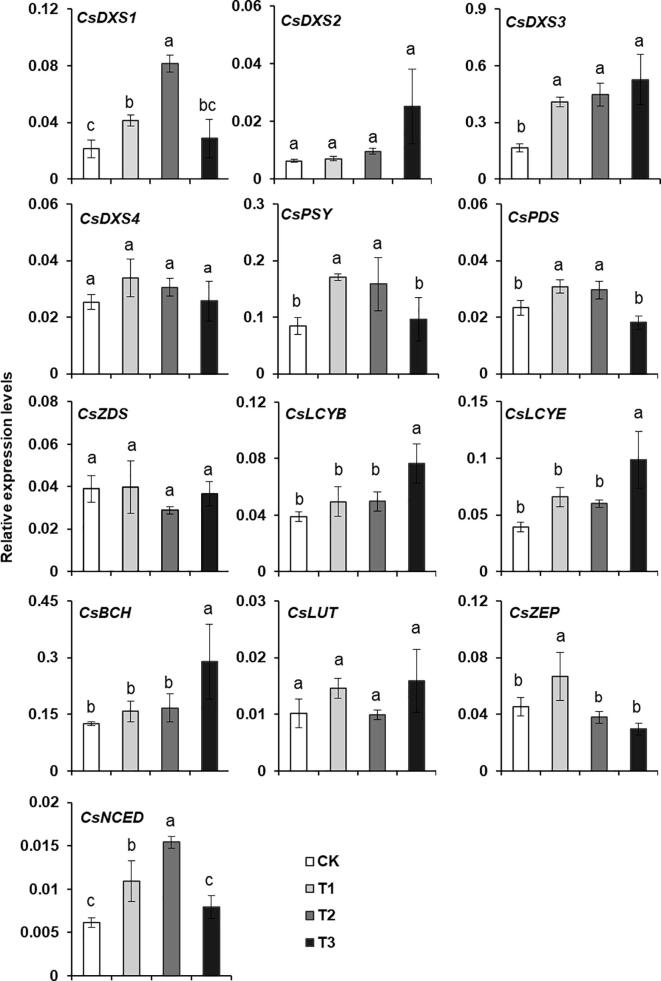
Expression level analysis of carotenoid biosynthetic genes of tea leaves under shading treatment for 14 days. CK, in nature light; T1, under 90% shading treatment; T2, under 98% shading treatment; T3, under 99.5% shading treatment. Data show mRNA levels relative to *CsEF1α* mRNA and are expressed as means ± S.D. (n = 3). Different letters above bars indicate significant differences using Duncan’s tests (*P* ≤ 0.05).

The transcription levels of the genes in the MEP pathway (*CsDXS1*–*4*) and the carotenoid biosynthesis pathway (*CsPSY*, *CsPDS*, *CsZDS*, *CsLCYB*, *CsLCYE*, *CsBCH*, *CsECH*, and *CsZEP*) were analyzed after the shading treatment of tea plants (Fig. 2). According to Duncan’s test, the expression levels of *CsDXS1*, *CsDXS3*, and *CsPSY* were shown to be significantly higher in T1 (90%) and T2 (98%) treatments than in control (CK). The *CsLCYB*, *CsLCYE*, and *CsBCH* expression levels also increased after shading treatments, but the increase was significant only for the 99.5% shading treatment (Fig. 2). These findings suggested that shading leads to carotenoid accumulation possibly because it up-regulates the expression of MEP pathway and carotenoid synthesis pathway genes.

### 3.2 Long-term dark treatment decreased the accumulation of carotenoids in tea leaves

**Fig. 3:**
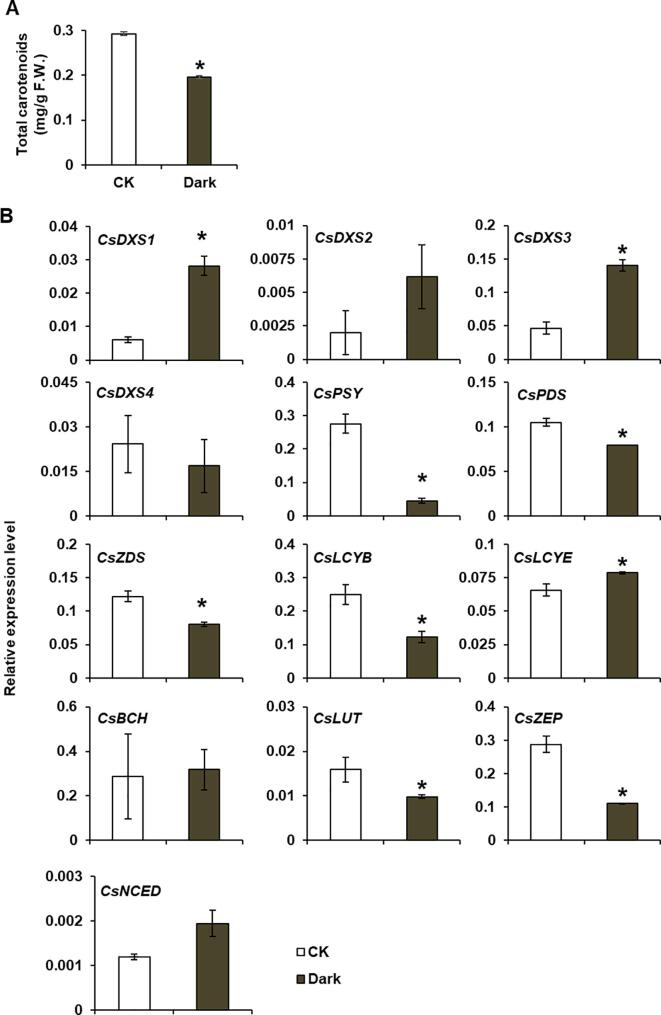
Analysis of carotenoids content (A) and carotenoid biosynthetic gene expression levels (B) of tea leaves under dark treatment for 14 days. CK, light intensity with 4000 lx, light/dark cycles (16 h/8h). Dark, continuous darkness environment. F.W., fresh weight. Data are expressed as means ± S.D. (n = 3). * *P* ≤ 0.05 using Student’s *t* test.

Many environmental factors are affected by shading treatment, including light intensity, light quality, and temperature. To study whether light represses or induces carotenoid accumulation in tea leaves, tea seedlings underwent long-term exposure to darkness for the subsequent analysis of the carotenoid accumulation in leaves. The resulting data revealed that the 14-day dark treatment significantly decreased the carotenoid content (Fig. 3A) as well as the expression levels of carotenoid biosynthesis genes (i.e., *CsPSY*, *CsPDS*, *CsZDS*, *CsLCYB*, *CsLUT*, and *CsZEP*) (Fig. 3B). The darkness-induced *CsDXS* expression patterns were similar to the expression patterns observed following the 14-day shading treatment, with *CsDXS1* and *CsDXS3* transcription up-regulated by darkness. These results reflect the complex regulatory effects of light on carotenoid synthesis and accumulation in tea plants under different light conditions. Long-term shading and long-term exposure to darkness differentially regulate the expression of carotenoid biosynthetic genes in tea leaves, but may similarly affect *CsDXS1* and *CsDXS3* transcription. Although the long-term incubation in darkness induced the MEP pathway, it significantly decreased the expression of carotenoid biosynthetic genes, suggesting that the carotenoid biosynthesis pathway may be the limiting factor for carotenoid accumulation under long-term dark treatment.

### 3.3 Key carotenoid-related enzymes in plastids and their functions

**Fig. 4:**
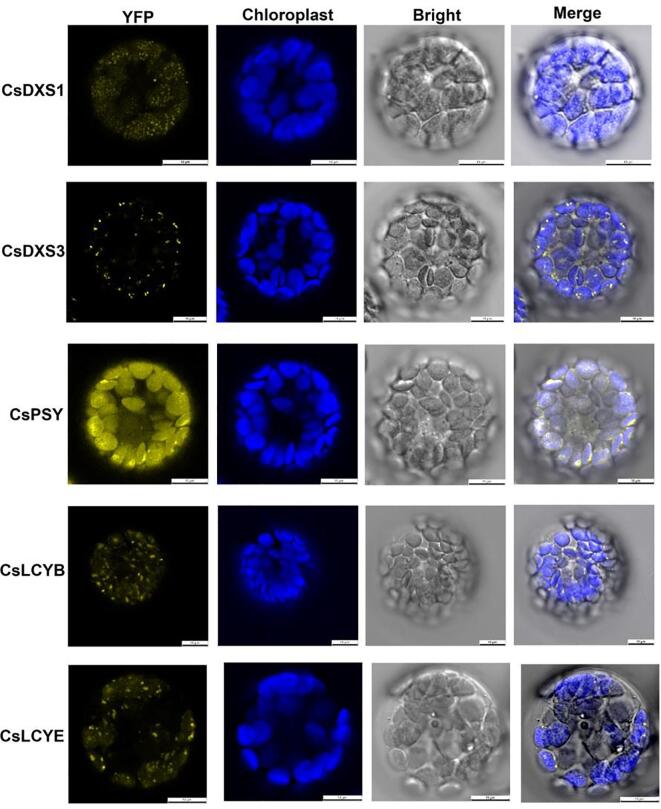
Subcellular location of carotenoid biosynthetic enzymes (CsDXS1, CsDXS3, CsPSY, CsLCYB and CsLCYE) in *Arabidopsis* protoplasts. Yellow fluorescence indicated CsDXS1/DXS3/PSY/LCYB/LCYE-YFP fusion proteins. Blue showed the chloroplast auto-fluorescence. Merged panel showed overlay of yellow, blue fluorescence and bright images. Bar = 10 μm. (For interpretation of the references to colour in this figure legend, the reader is referred to the web version of this article.)

Carotenoids are synthesized and accumulate in plant cell plastids. Accordingly, the enzymes encoded by carotenoid biosynthesis pathway genes are likely localized to plastids. First, on the basis of their full-length sequences, we predicted the subcellular localizations of CsDXS1, CsDXS3, CsPSY, CsLCYB, and CsLCYE using TargetP. The results indicated that all of these enzymes are located in plastids. The predicted subcellular localizations were verified by an *A. thaliana* protoplast transformation experiment. The coding sequences of the analyzed enzymes were inserted into the YFP vector. The recombinant plasmids were then transferred into *A. thaliana* protoplasts. The subcellular localization of each enzyme was determined according to the detected fluorescence in the protoplasts. Consistent with the TargetP online predictions, the protoplast examinations indicated that the enzymes were present in *A. thaliana* chloroplasts (Fig. 4).

**Fig. 5:**
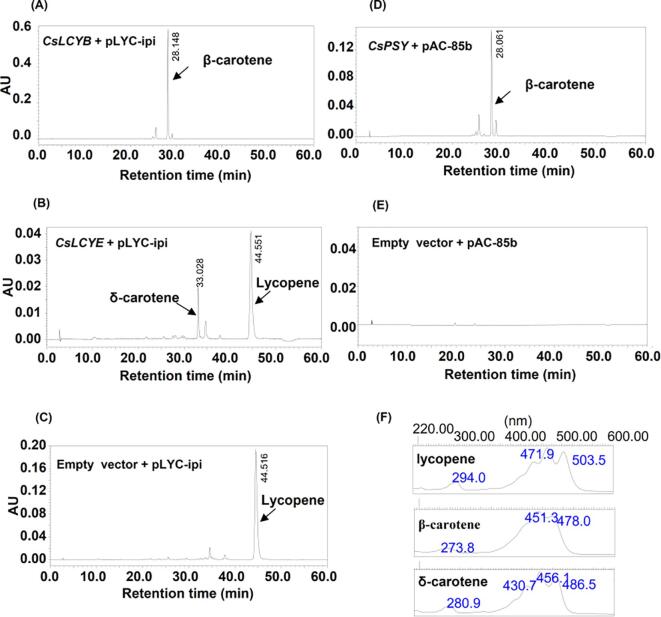
Functional analysis of enzymes expressed in *E. coli* strains. Functional analysis of lycopene β-/ε-cyclase activity expressed in *E. coli* lycopene-complementation strains (A-C). Functional analysis of CsPSY in functional complementaion of carotenoid synthesis in *E. coli* (D and E). Pigments extracted from *E. coli* DH5α cells harboring plasmids pAC-LYC and CsLCYB (A); Plasmids pAC-LYC and CsLCYE (B); Empty vector and plasmids pAC-LYC (C); pAC-85b and CsPSY (D); Empty vector and pAC-85b (E). The absorption spectra of lycopene, β-carotene, and δ-carotene (F).

Functional analyses of lycopene cyclases were conducted in a heterologous *E. coli* system. Briefly, *E*. *coli* DH5α cells harboring a pAC-LYC plasmid were transformed with expression plasmids containing *CsLCYB* or *CsLCYE*. Carotenoids extracted from the bacterial cells were analyzed by HPLC (Fig. 5). Cells harboring pAC-LYC and the empty vector produced only lycopene (Fig. 5C); however, β-carotene was produced following the addition of plasmid containing *CsLCYB* (Fig. 5A), suggesting that CsLCYB was able to convert the lycopene produced by the cells to β-carotene. When *CsLCYE* was expressed in cells harboring pAC-LYC, monocyclic δ-carotene and lycopene were detected, implying that CsLCYE is a functional lycopene ε-cyclase. The presence of lycopene in the cells (Fig. 5B) suggested that the catalytic efficiency of CsLCYE was too low to convert all of the substrate to monocyclic δ-carotene *in vitro*. To determine whether CsPSY can mediate carotenoid synthesis in a bacterial system, the ORF of *CsPSY* was subcloned into the *E. coli* expression vector for a co-transformation with the pAC-85b plasmid, which contains all of the coding sequences necessary for carotenoid biosynthesis, except for *PSY*. The product (β-carotene) was detected in bacterial cells transformed with the plasmid carrying *CsPSY* (Fig. 5D), but not in the cells transformed with the empty vector (Fig. 5E).

**Fig. 6:**
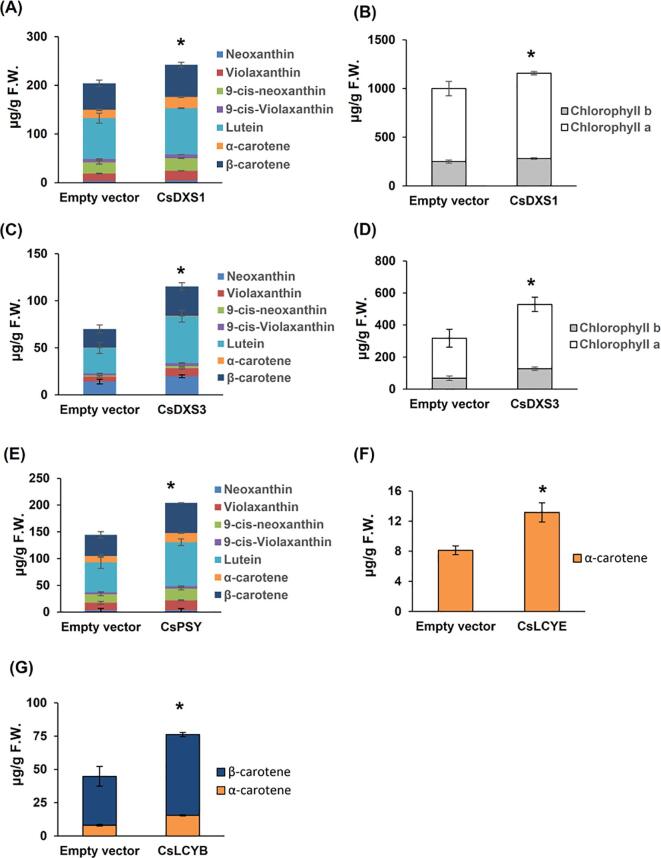
Functional analysis of enzymes using transient overexpression in tobacco leaves. (A) Carotenoid content and composition in tobacco leaves with overexpressed *CsDXS1* gene compared to empty vector. (B) Chlorophyll content and composition in tobacco leaves with overexpressed *CsDXS1* gene compared to empty vector. (C) Carotenoid content and composition in tobacco leaves with overexpressed *CsDXS3* gene compared to empty vector. (D) Chlorophyll content and composition in tobacco leaves with overexpressed *CsDXS3* gene compared to empty vector. (E) Carotenoid content and composition in tobacco leaves with overexpressed *CsPSY* gene compared to empty vector. (F) α-Carotene content in tobacco leaves with overexpressed *CsLCYE* gene. (G) α-Carotene and β-carotene contents in tobacco leaves with overexpressed *CsLCYB* gene. F.W., fresh weight. Data are expressed as means ± S.D. (n = 3). * *P* ≤ 0.05 using Student’s *t* test based on the total carotenoid contents.

To investigate the CsDXS1, CsDXS3, CsLCYB, CsLCYE, and CsPSY functions *in vivo*, the corresponding genes fused to the GFP-encoding sequence were transiently overexpressed in *N. benthamiana* plants (Fig. 6). The carotenoid and chlorophyll contents were significantly higher in the *N. benthamiana* cells overexpressing *CsDXS1* or *CsDXS3* than in the control cells (i.e., those with the empty vector) (Fig. 6A–D). The overexpression of *CsPSY* in *N. benthamiana* cells led to a noteworthy increase in the carotenoid contents (relative to the control levels) (Fig. 6E). Increased α-carotene was detected in the *CsLCYE-*overexpressing *N. benthamiana* cells (Fig. 6F). The overexpression of *CsLCYB* in *N. benthamiana* cells resulted in significant increases in the α-carotene and β-carotene contents (Fig. 6G). These results indicated that all of the analyzed enzymes are responsible for the carotenoid accumulation in tea plants.

## 4 Discussion

### 4.1 Shade tolerance of tea plants and carotenoid accumulation under shading conditions

Plant growth depends on light. We previously demonstrated that a long-term incubation (e.g., 14 days) in darkness leads to the yellowing of tea leaves, the collapse of the chloroplast structure, and the degradation of Rubisco, which is accompanied by an increase in the free amino acid content (Chen et al., 2017). In the study, the carotenoid pigment contents declined significantly in the long-term absence of light (Fig. 3A). Compared with the plants treated with natural light intensity, the tea plants that underwent a 14-day shading treatment or a heavy shading (99.5%) treatment had higher leaf carotenoid contents. The differences in the effects of the long-term shading and long-term exposure to darkness on the carotenoid accumulation pattern indicate that under long-term light stress conditions, light is not a simple enhancer or inhibitor of carotenoid accumulation in tea plants. Hence, the regulatory mechanism underlying the effects of light on tea plants is complex.

In the current study, black shading nets were used to decrease the light intensity during the long-term shading treatment. The treated tea plants lacked a typical shade-avoidance response, but exhibited traits associated with shade-tolerant plants (e.g., shortened stems and increased chlorophyll and carotenoid contents) (Fig. 1). The phenomenon is in accord with the finding of a recent study, which confirmed that a shading treatment of tea plants stimulates the expression of genes involved in chloroplast development, optimizes the chloroplast structure, and increases the chlorophyll content (Liu et al., 2020). Therefore, the observed increase in the chlorophyll and carotenoid contents may be a tea plant adaptive response to long-term shading. This phenomenon is also similar to the findings of previous investigations on cassava, yam, sweet potato, and taro, which revealed that the leaf chlorophyll and carotenoid contents are significantly higher in plants shaded in the field than in control plants grown under natural conditions (Johnston & Onwueme, 1998). Accordingly, the physiological response of these shade-tolerant plants to long-term shading differs from that of *A. thaliana*. They may be able to increase their light capture capacity by increasing the chlorophyll and carotenoid contents to adapt to increasing durations of low-light conditions.

### 4.2 Regulation of carotenoid accumulation in shade-tolerant tea plants

Studies on the regulation of carotenoid levels by shading have primarily focused on the shade-avoiding plant *A. thaliana* (Bou-Torrent et al., 2015). The mechanism regulating carotenoid accumulation in shade-tolerant plants remains relatively uncharacterized. The shade-regulated expression of carotenoid genes in tea plants has been reported for light-sensitive tea cultivars (Li et al., 2016, Song et al., 2017). These studies revealed that the shade-induced up-regulated expression of genes related to carotenoid biosynthesis (i.e., *PSY*, *PDS*, *ZDS*, *LCYE*, *LCYB*, *ZEP*, *VDE*, *BCH*, and *ECH*) shows a positive correlation with carotenoid contents. In this study, we assessed the effects of a long-term shading treatment on the expression of MEP pathway and carotenoid synthesis pathway genes in the green tea variety ‘Jinxuan’. It was found that shading increased the expression of most examined genes, including key genes (*CsDXS1*, *CsDXS3*, *CsPSY*, *CsLCYB*, and *CsLCYE*).

A tobacco transient overexpression system was used for the functional characterization of DXS in tea plants. The overexpression of *CsDXS1* and *CsDXS3* in tobacco leaves enhanced chlorophyll and carotenoid accumulation. Thus, high *CsDXS1* and *CsDXS3* expression levels in shaded tea plants may contribute to increases in chlorophyll and carotenoid levels. The *DXS* gene encodes a rate-limiting enzyme in the MEP synthesis pathway, which has multiple *DXS* homologs of genes in many plants, but only one member in *A. thaliana*. These genes reportedly affect carotenoid synthesis (Cordoba et al., 2011, Cordoba et al., 2009, Ruiz-Sola and Rodríguez-Concepción, 2012). The effects of the MEP pathway on chlorophyll and carotenoid synthesis may be regulated by light (Hsieh & Goodman, 2005). In *A. thaliana*, *DXS1* expression is down-regulated by the PIF1 transcription factor in darkness, whereas it is up-regulated by the HY5 transcription factor under light, suggesting that *DXS* expression may be regulated by light. In the present study, the *CsDXS1* and *CsDXS3* expression levels were significantly up-regulated in tea plants by the long-term shading and long-term incubation in darkness (Fig. 2, Fig. 3B). Combined with the results of the transient gene overexpression in tobacco cells, these findings suggest that CsDXS1 and CsDXS3 may be important for the accumulation of carotenoids in shaded tea plants. During the long-term exposure to darkness a significant decrease in the expression of the gene encoding the first rate-limiting enzyme (CsPSY) (Fig. 3B) might explain the decrease in carotenoid contents. Additionally, whether the transcriptional regulation of *CsDXS1* and *CsDXS3* in tea leaves during long-term shading or darkness is similar to the transcriptional regulation under natural day/night conditions should be investigated in future studies.

The induction of the shade-avoidance response of *A. thaliana* and other plants by a low red:far-red light ratio causes the leaf carotenoid contents to decrease. Phytochrome PIFs and the transcription cofactor PHYTOCHROME-RAPIDLY REGULATED1 are transcriptional regulators. The PIF proteins (excluding PIF7) can inhibit *PSY* expression under normal and reduced light conditions (Bou-Torrent et al., 2015). However, unlike the shade-avoidance response of *A. thaliana*, the shading of tea plants resulted in increased carotenoid contents and up-regulated *CsPSY* expression, implying the mechanism regulating *CsPSY* expression varies between tea and *A. thaliana*. This potential difference between plant species will need to be experimentally verified. We also observed that the *CsLCYB* and *CsLCYE* expression levels in tea plants were up-regulated after the shading treatment. A study indicated that CpbHLH1 helps regulate *CpLCYB* expression in developing papaya fruit (Zhou et al., 2019). Another recent investigation demonstrated that the CsMADS5 transcription factor can directly regulate *LCYb1* expression during citrus fruit development (Lu, Ye, Zhu, Zhang, Zhang, Xu, & Deng, 2021). The transcription factors CpEIN3a and CpNAC2 were found to be involved in regulating the expression of *CpLCYE* during papaya fruit development (Fu, Han, Kuang, Chen, & Lu, 2017). Although the light-related regulatory factors controlling *LCYB* and *LCYE* expression in plants have not been identified, the transcription of the corresponding tea genes may be regulated by long-term shading or darkness (Fig. 2, Fig. 3B). Thus, they are very likely regulated by a light-related transcription factor.

## 5 Conclusions

In this study, long-term shading (90%–95%) induced the accumulation of carotenoids in tea plants, whereas long-term incubation in darkness had the opposite effect. To explore the influence of shading on carotenoid accumulation in tea plants, the expression levels of MEP pathway and carotenoid biosynthesis pathway genes were analyzed after a shading treatment. Shading mainly up-regulated the *CsDXS1*, *CsDXS3*, *CsPSY*, *CsLCYB*, and *CsLCYE* transcription levels. The absence of light significantly inhibited the expression of most genes in the carotenoid synthesis pathway. The subcellular localization and functional analyses of CsDXS1, CsDXS3, CsPSY, CsLCYB, and CsLCYE suggested that these enzymes may be crucial for the increase of carotenoids in tea leaves during a long-term shading treatment. Therefore, the results of this study will help to elucidate the molecular mechanism of carotenoid accumulation in shaded tea plants and may help tea producers modify their cultivation practices to optimize tea plant quality-related traits.

## Author contributions

Z.Y. proposed the project, conceived and designed the experiments, designed the manuscript outline and revised the manuscript, and supported the project. X.F. designed the experiments, contributed to analyses of metabolites and gene expression, enzyme function identification, analyzed the results and drew the figures, and wrote the manuscript. J.C. contributed to shading experiments in tea field and indoor experiments. J.L. contributed to shading experiments in tea field. G.D. contributed to subcellular localization investigation. J.T. provided tea plant resources. All authors reviewed the manuscript.

## Declaration of Competing Interest

The authors declare that they have no known competing financial interests or personal relationships that could have appeared to influence the work reported in this paper.
