# Supplementary material for: Mechanism underlying the carotenoid accumulation in shaded tea leaves
Source: Food Chem X. 2022 May 4;14:100323. doi: 10.1016/j.fochx.2022.100323 (PMC9097638; doi:10.1016/j.fochx.2022.100323)
Supplement: Supplementary data 1 [file mmc1.doc]

**Running title:** Carotenoid accumulation in shaded tea leaves

**Title:** Mechanism underlying the carotenoid accumulation in shaded tea leaves

**Authors：**Xiumin Fu ^a^, Jiaming Chen ^a, b^, Jianlong Li ^c^, Guangyi Dai ^a^, Jinchi Tang ^c^, Ziyin Yang ^a, b, d^, *

**Affiliation:**

*^a^ Guangdong Provincial Key Laboratory of Applied Botany & Key Laboratory of South China Agricultural Plant Molecular Analysis and Genetic Improvement, South China Botanical Garden, Chinese Academy of Sciences, No. 723 Xingke Road, Tianhe District, Guangzhou 510650, China*

*^b^ University of Chinese Academy of Sciences, No. 19A Yuquan Road, Beijing 100049, China*

*^c^ Tea Research Institute, Guangdong Academy of Agricultural Sciences & Guangdong Provincial Key Laboratory of Tea Plant Resources Innovation and Utilization, No. 6 Dafeng Road, Tianhe District, Guangzhou 510640, China*

*^d^ Center of Economic Botany, Core Botanical Gardens, Chinese Academy of Sciences, No. 723 Xingke Road, Tianhe District, Guangzhou 510650, China*

^*^ **Corresponding author:**

Ziyin Yang, South China Botanical Garden, Chinese Academy of Sciences, Xingke Road 723, Tianhe District, Guangzhou 510650, China; Tel: +86-20-38072989; Email address: zyyang@scbg.ac.cn.

**Supporting information**

**Table S1** qRT-PCR analysis primers used in this study

| **Gene** | **Forward primer** | **Reverse primer** | **Locus identifier in TPIA database** |
| --- | --- | --- | --- |
| *CsDXS1* | TAAGGGAAGGAAGTAGGGT | ACAGTGATTGAGATGCCAA | TEA008876 |
| *CsDXS2* | GGGATATGGGTCAATAGTT | TAAGATTTCATGCTCCTTC | TEA026768 |
| *CsDXS3* | TAGATGGGCTTCTTGATGGC | ACTGTTGCGGCAATATGAG | TEA012941 |
| *CsDXS4* | TCTGGATGGGCTTCTTGAT | ACTGTCACCCTCAGCGTTT | TEA012756 |
| *CsPSY* | GCTCGATGCTGCTTTGTCAG | AGATTCAGGCGCAATTCCCA | TEA022104 |
| *CsPDS* | AAGGACGTGTGCCCTTTGAA | CTGCACCAGCAATGACAACC | TEA016017 |
| *CsZDS* | GATACAACGGCTGGGTCACA | AGCAATGAGCCTTGTCCCTC | TEA007854 |
| *CsLCYE* | CGATCACTGTCAGAGGCTCC | CTTGCGGCCAAAGAGTGTTC | TEA013706 |
| *CsLCYB* | GACACTACCTGGTCTGGTGC | AAGAACTACGGCGGCTTGAA | TEA012946 |
| *CsLUT* | GGCTTGTTGCTGAGGTCTCT | AGCAGAGCCATTGAGTGCAT | TEA014070 |
| *CsZEP* | TGCTCTTGAGCGTGCTGTTA | GTTCGCTCCGCAAATCTGTC | TEA013909 |
| *CsVDE* | CGAATACGGACTCCAGACGG | GCATCGTTCCTGCCTCGATA | TEA013090 |
| *CsNCED* | CAATGGGTTTGCTCCGTTCC | ATCCGTCATCGTTGTCTCCG | TEA022237 |
| *CsBCH* | GCACGAGTCTCACCATAAACC | ACTCATCAAGCCCTCCCACT | TEA031348 |

**Table S2** Primers for the full length gene cloning

| **Gene** | **Forward primer** | **Reverse primer** |
| --- | --- | --- |
| *CsDXS1* | ATGGGTTCTTGTGGTGTTATC | CTAGAGGTTGAGAAGGTGAAGGT |
| *CsDXS3* | ATGGCTCTTTGTACGTCCTCA | CTATGTCATGATCTCTAGAGCCT |
| *CsPSY* | ATGTCTGCAGCTCTGTTATGGG | CTATGACGACTTTGCCGAAAGC |
| *CsLCYE* | ATGGAGTGTATCGGAGCTCG | CTATATTGTGAGATATGTTCT |
| *CsLCYB* | ATGGATACTTTGCTCAAGATTC | TTATTCCCTATCCTGTACTAAG |

**Table S3 Primers for pSAT6-EYFP-N1 vector construction**

| **Gene** | **Forward primers** | **Reverse primers** |
| --- | --- | --- |
| *CsDXS1* | CTCAAGCTTCGAATTCATGGGTTCTTGTGGTGTTATCAAG | CCATCAGGATCCCGGGGAGGTTGAGAAGGTGAAGGTTTTCC |
| *CsDXS3* | CTCAAGCTTCGAATTCATGGCTCTTTGTACGTCCTC | CCATCAGGATCCCGGGTGTCATGATCTCTAGAGCCTTTC |
| *CsLCYB* | CTCAAGCTTCGAATTCATGGATACTTTGCTCAAGATTCA | CCATCAGGATCCCGGGTTCCCTATCCTGTACTAAGTTGT |
| *CsLCYE* | CTCAAGCTTCGAATTCATGGAGTGTATCGGAGCT | CCATCAGGATCCCGGGTATTGTGAGATATGTTCTTATCAT |
| *CsPSY* | CTCAAGCTTCGAATTCATGTCTGCAGCTCTGTTATGGG | CCATCAGGATCCCGGGTGACGACTTTGCCGAAAGC |

**Table S4 Primers for *E*. *coli*. expression vector construction**

| **Gene** | **Forward primers (5' to 3')** | **Reverse primers (5' to 3')** |
| --- | --- | --- |
| *CsLCYB* | CTCAAGCTTCGAATTCATGGATACTTTGCTCAAGATTCA | CCATCAGGATCCCGGGTTCCCTATCCTGTACTAAGTTGT |
| *CsLCYE* | CTCAAGCTTCGAATTCATGGAGTGTATCGGAGCT | CCATCAGGATCCCGGGTATTGTGAGATATGTTCTTATCAT |
| *CsPSY* | CTCAAGCTTCGAATTCATGTCTGCAGCTCTGTTATGGG | CCATCAGGATCCCGGGTGACGACTTTGCCGAAAGC |

**Table S5** Primers for vectors of transient overexpression in tobacco

| **Genes** | **Forward primers** | **Reverse primers** |
| --- | --- | --- |
| *CsDXS1* | CGGTACCCGGGGATCCATGGGTTCTTGTGGTGTTATCAAG | CCATGGTGGCACTAGTGAGGTTGAGAAGGTGAAGGTTTTCC |
| *CsDXS3* | CGGTACCCGGGGATCCATGGCTCTTTGTACGTCCTC | CCATGGTGGCACTAGTTGTCATGATCTCTAGAGCCTTTC |
| *CsPSY* | CGGTACCCGGGGATCCATGTCTGCAGCTCTGTTATGGG | CCATGGTGGCACTAGTTGACGACTTTGCCGAAAGC |
| *CsLCYB* | CGGTACCCGGGGATCCATGGATACTTTGCTCAAGATTCA | CCATGGTGGCACTAGTTTCCCTATCCTGTACTAAGTTGT |
| *CsLCYE* | CGGTACCCGGGGATCCATGGAGTGTATCGGAGCT | CCATGGTGGCACTAGTTATTGTGAGATATGTTCTTATCAT |


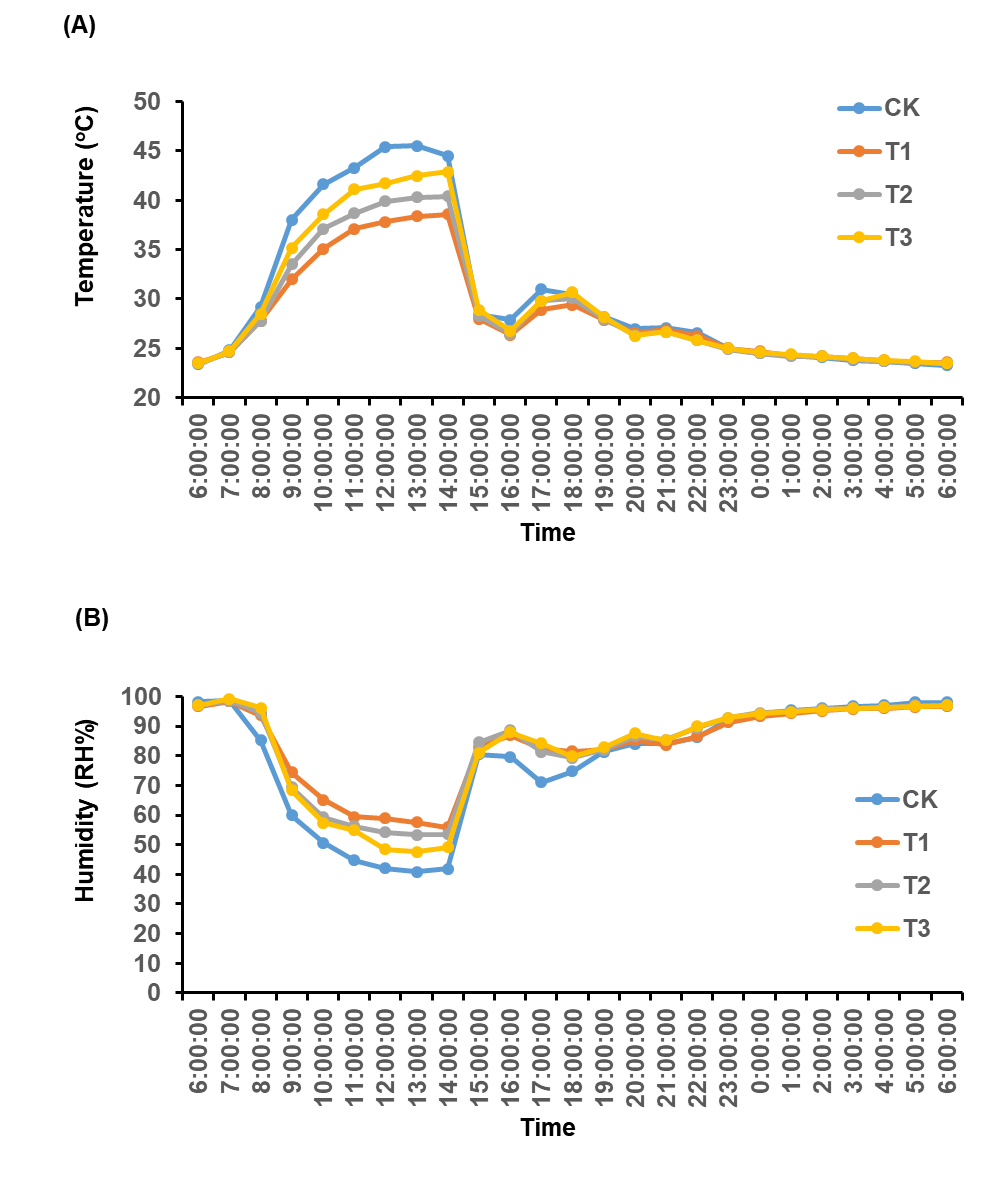


**Figure S1** The temperature (A) and humidity (B) during day and night in the tea field experiment.

CK, in nature light; T1, under 90% shading treatment; T2, under 98% shading treatment; T3, under 99.5% shading treatment.


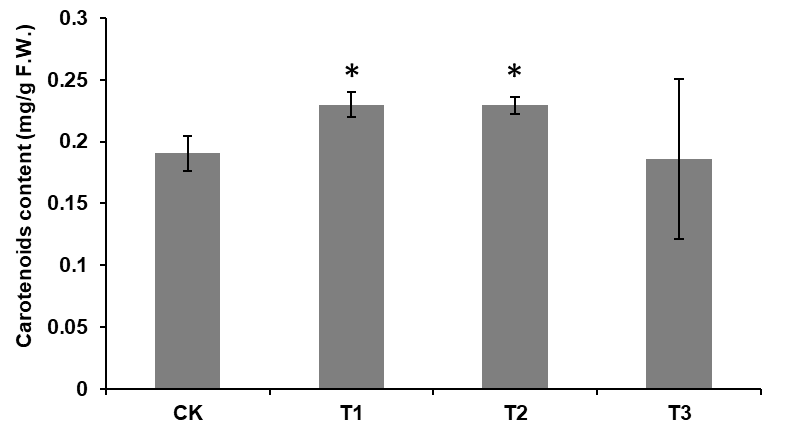


**Figure S2** Carotenoid content analysis of tea leaves under shading treatment for 7 days

CK, in nature light; T1, under 90% shading treatment; T2, under 98% shading treatment; T3, under 99.5% shading treatment. Data show the total carotenoid levels and are expressed as means ± S.D. (n = 3). Turkey test was carried out to analysis of the differences in the data. * indicates the significant difference between the treatment compared to the control (*P*≤0.05).
